# Retinal Occlusive Vasculitis in a Patient with Hyperimmunoglobulin E Syndrome

**DOI:** 10.1155/2021/6317358

**Published:** 2021-12-23

**Authors:** Mohsen Farvardin, Mohammad Hassan Jalalpour, Mohammad Reza Khalili, Golnoush Mahmoudinezhad, Fereshteh Mosavat, Soheila Aleyasin, Hamidreza Jahanbani-Ardakani

**Affiliations:** ^1^Poostchi Ophthalmology Research Center, Department of Ophthalmology, School of Medicine, Shiraz University of Medical Sciences, Shiraz, Iran; ^2^Hamilton Glaucoma Center, Shiley Eye Institute, Viterbi Family Department of Ophthalmology, University of California San Diego, La Jolla, CA, USA; ^3^Division of Allergy and Immunology, Department of Pediatrics, School of Medicine, Shiraz University of Medical Sciences, Shiraz, Iran; ^4^Department of Ophthalmology, School of Medicine, Shiraz University of Medical Sciences, Shiraz, Iran; ^5^Student Research Committee, Shiraz University of Medical Sciences, Shiraz, Iran

## Abstract

**Background:**

Hyperimmunoglobulin E syndrome (HIES), or Job's syndrome, is a primary immunodeficiency disorder that is characterized by an elevated level of IgE with values reaching over 2000 IU (normal < 200 IU), eczema, and recurrent staphylococcus infection. Affected individuals are predisposed to infection, autoimmunity, and inflammation. Herein, we report a case of HIES with clinical findings of retinal occlusive vasculitis. *Case Presentation*. A 10-year-old boy with a known case of hyperimmunoglobulin E syndrome had exhibited loss of vision and bilateral dilated fixed pupil. Fundoscopic examination revealed peripheral retinal hemorrhaging, vascular sheathing around the retinal arteries and veins, and vascular occlusion in both eyes. A fluorescein angiography of the right eye showed hyper- and hypofluorescence in the macula and hypofluorescence in the periphery of the retina, peripheral arterial narrowing, and arterial occlusion. A fluorescein angiography of the left eye showed hyper- and hypofluorescence in the supranasal area of the optic disc. Macular optical coherence tomography of the right eye showed inner and outer retinal layer distortion. A genetic study was performed that confirmed mutations of the dedicator of cytokinesis 8 (DOCK 8). HSV polymerase chain reaction testing on aqueous humor and vitreous was negative, and finally, the patient was diagnosed with retinal occlusive vasculitis.

**Conclusion:**

Occlusive retinal vasculitis should be considered as a differential diagnosis in patients with hyperimmunoglobulin E syndrome presenting with visual loss.

## 1. Introduction

Hyperimmunoglobulin E syndrome (HIES), or Job's syndrome, is a primary immunodeficiency disorder. It was first described in 1966 by Davis and is characterized by an elevated level of IgE with values reaching over 2000 IU (normal < 200 IU), eczema, and recurrent staphylococcus infection. Affected individuals are predisposed to infection, autoimmunity, and inflammation [1, 2]. Herein, we report a case of HIES with clinical findings of retinal occlusive vasculitis.

## 2. Case Presentation

A 10-year-old boy with a known case of HIES was referred to the pediatric ophthalmology clinic at Poostchi Eye Clinic, which is affiliated with Shiraz University of Medical Science in Shiraz City in Iran. He had exhibited loss of vision and bilateral dilated fix pupils about 1 month before visiting the clinic. His family history revealed that his sister had also been diagnosed with HIES and had succumbed to lymphoma. The patient had been diagnosed with AR-HIES in early childhood. He had presented with eczema, severe dermatitis, recurrent skin abscesses, herpes simplex virus (HSV), gingivostomatitis, and recurrent sinopulmonary infections. He also exhibited a high serum IgE level (600 IU/ml). The NIH hyper IgE score was 40 [3].

**Figure 1:**
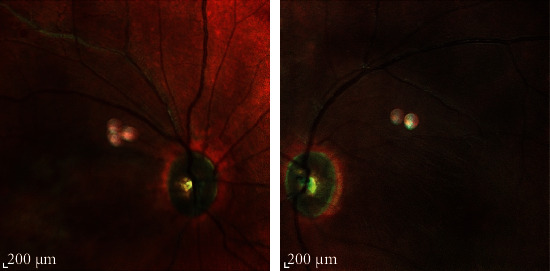
Multicolor SLO: (OD) optic nerve pallor, intraretinal hemorrhages, areas of macular whitening, perivascular infiltration, and retinal vein occlusion (thread like); (OS) optic nerve pallor, macular nerve fiber loss, intraretinal hemorrhages, and areas of macular whitening.

**Figure 2:**
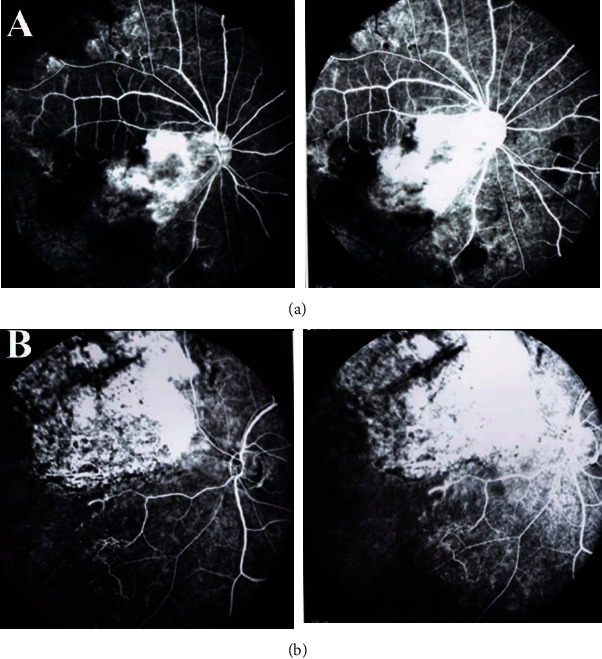
(a) Fundus fluorescein angiography of the right eye shows hyper- and hypofluorescence in the macular area in favor of retinal hemorrhage and vascular leakage and peripheral arterial narrowing and occlusion. (b) Fundus fluorescein angiography of the left eye shows hyper- and hypofluorescence that is in favor of vascular leakage, retinal hemorrhage, and ischemia in the supranasal area of the optic disc.

**Figure 3:**
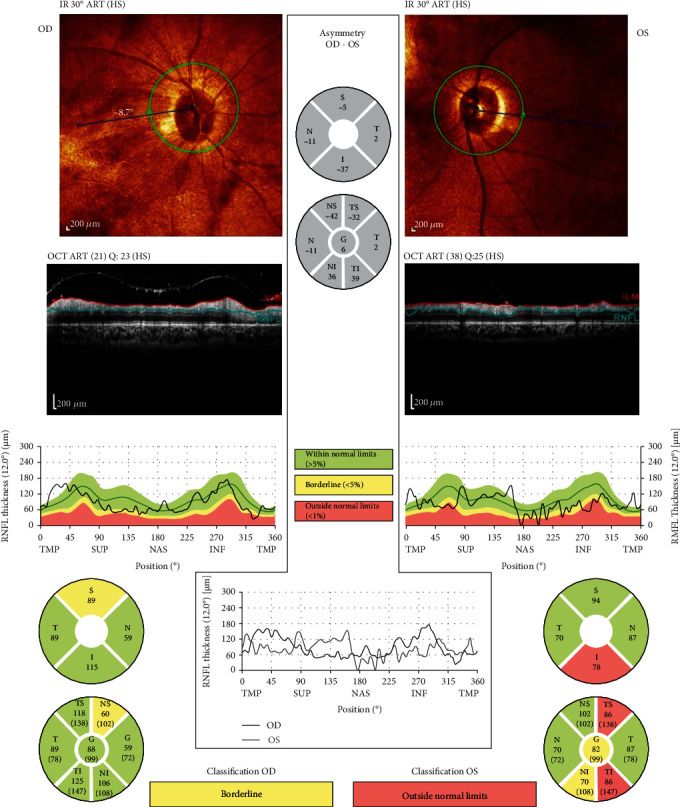
OCT of optic nerves (NFL analysis): (OD) mild NFL damage, especially at superior part; (OS) more severe NFL damage, especially at inferior part.

**Figure 4:**
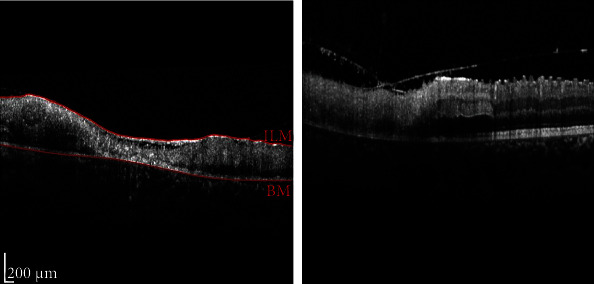
OCT of macula: (OD) faint epiretinal membrane, inner and outer retinal layers' distortion with some atrophic areas, and areas of subretinal hyporeflectivity (fluid); (OS) epiretinal membrane, intraretinal hyperreflectivity (hemorrhage), and subretinal hyporeflectivity (fluid).

An ophthalmic physical examination showed visual acuity of 20/200 in the right eye (OD) and 20/400 in the left eye (OS). Slit lamp examination of both eyes revealed severe meibomian gland dysfunction, blepharitis, and fixed dilated pupils. Funduscopic examination revealed optic nerve pallor, intraretinal hemorrhage, areas of macular whitening, perivascular infiltration, and retinal vein occlusion. (Figure 1). Fluorescein angiography of the right eye showed hyper- and hypofluorescence that is in favor of vascular leakage and retinal hemorrhage in the macula and hypofluorescence in the periphery of the retina, peripheral arterial narrowing, and arterial occlusion. Fluorescein angiography of the left eye showed hyper- and hypofluorescence that is in favor of vascular leakage and retinal hemorrhage and ischemia in the supranasal area of the optic disc (Figure 2). Optical coherence tomography (OCT) of optic nerves (NFL analysis) showed retinal nerve fiber damage (NFL) in both eyes (Figure 3). Macular optical coherence tomography of the macula showed epiretinal membrane, inner and outer retinal layers' distortion with some atrophic areas, areas of subretinal hyporeflectivity (fluid), and intraretinal hyperreflectivity (hemorrhage) (Figure 4).

Magnetic resonance imaging of the brain with and without contrast revealed the prominence and dilatation of the cerebral ventricles (lateral and 3rd and 4th ventricles) with possible communicating hydrocephalus, generalized cortical atrophy, and pansinusitis. Magnetic resonance angiography of the brain revealed irregular borders of both posterior cerebral arteries, possible vasculitis, and prominent central and peripheral CSF spaces, suggesting atrophy. A genetic study was performed that confirmed homozygous mutations of the dedicator of cytokinesis 8 (DOCK 8).

To rule out HSV infection, we performed anterior chamber and vitreous tap. The specimen was tested by the microbiological laboratory for HSV PCR and the result was negative. The patient was diagnosed with occlusive retinal vasculitis. Panretinal photocoagulation (PRP) laser treatment was performed to treat the retinal ischemia.

## 3. Discussion and Conclusion

The majority of HIES cases are sporadic; however, autosomal dominant (AD) and autosomal recessive (AR) inherited types have been described in the literature [1]. In 60-70 percent of the AD type, mutation occurs in the STAT3 [4]. In the AR type, which is characterized by persistent viral skin infections and mucocutaneous candidiasis, mutation occurs in the dedicator of cytokinesis 8 (DOCK8) in many cases [4, 5]. DOCK8 mutation-related vasculitis has been previously reported [6].

The prevalence of vascular abnormalities in HIES is unknown. In patients with HIES, vascular abnormalities of the brain, vein, skin, lung, aorta, heart, and foot have been reported [1]. Other ocular manifestations of HIES have been described, including conjunctivitis [7], keratitis, corneal perforation, xanthelasma, undefined eye lid nodules, giant chalazia, strabismus, and retinal detachment with complicated cataracts and keratoconus [8, 9]. Retinal vasculitis has been described in common variable immunodeficiency [10]. Retinal hemorrhage is also reported as one of the ocular manifestations of hyperimmunoglobulin E syndrome [11]. In addition to vascular abnormality, our patient suffered from MGD, blepharitis, and fixed dilated pupils. The cause in that entity and in our case could be related to autoimmunity or to the occlusion from the high level of IgE as it can induce synthesis of the leukotrienes, chemokines, and cytokines leading to aggregation of the leukocytes and eosinophils with secondary occlusion, and in this setting, the use of omalizumab can potentially control this occlusive process [12].

Our patient was diagnosed with autosomal recessive HIES with DOCK8 mutation. This mutation is associated with a wide spectrum of vascular abnormalities caused by partial T cell deficiency and dysregulation of the immune system, ultimately leading to CNS and systemic vasculitis and lymphoma [13]. Fixed dilated pupils as presented in our patient may have been the result of pupillary sphincter neural damage induced by microangiopathic vasculitis. Central retinal occlusion has previously been reported in autosomal dominant HIES [7]. Our patient was autosomal recessive HIES with sheathing and occlusion of retinal vessels (retinal occlusive vasculitis).

In conclusion, occlusive retinal vasculitis should be considered as a differential diagnosis in patients with hyperimmunoglobulin E syndrome presenting with visual loss. A thorough ophthalmic examination can confirm the diagnosis.

## Data Availability

The data that support the findings of this study are available from the corresponding author, upon reasonable request.
